# Lack of Neuroprotective Effect of Celastrol Under Conditions of Proteasome Inhibition by Lactacystin in In Vitro and In Vivo Studies: Implications for Parkinson’s Disease

**DOI:** 10.1007/s12640-014-9477-9

**Published:** 2014-05-20

**Authors:** Jolanta Konieczny, Danuta Jantas, Tomasz Lenda, Helena Domin, Anna Czarnecka, Katarzyna Kuter, Maria Śmiałowska, Władysław Lasoń, Elżbieta Lorenc-Koci

**Affiliations:** 1 Department of Neuropsychopharmacology, Institute of Pharmacology, Polish Academy of Sciences, Smętna 12 St., 31-343 Kraków, Poland; 2 Department of Experimental Neuroendocrinology, Institute of Pharmacology, Polish Academy of Sciences, Smętna 12 St., 31-343 Kraków, Poland; 3 Department of Neurobiology, Institute of Pharmacology, Polish Academy of Sciences, Smętna 12 St., 31-343 Kraków, Poland

**Keywords:** Celastrol, Dopamine, Lactacystin, Parkinson’s disease, SH-SY5Y cells, Substantia nigra

## Abstract

A number of studies suggest that the ubiquitin–proteasome system (UPS) impairment may underlie neuronal death in Parkinson’s disease. Celastrol is a neuroprotective agent with anti-inflammatory and antioxidant properties. The aim of this study was to determine whether celastrol may exert neuroprotective effects both in vitro and in vivo under conditions of the lactacystin-induced UPS inhibition. In the in vitro study, mouse primary cortical neurons and neuroblastoma SH-SY5Y cells were incubated with lactacystin for 48 h (2.5 and 10 μg/ml, respectively). The animal study was performed on male Wistar rats injected unilaterally with lactacystin (5 μg/2 μl) into the substantia nigra (SN) pars compacta. In the in vitro study, we did not found any protective effects of celastrol, given either in the pre- or co-treatment mode. Moreover, in the higher concentrations, celastrol itself reduced cell viability, and enhanced the lactacystin-induced cell death in both types of cells. In the in vivo study, none of the celastrol doses (0.3–3 mg/kg) attenuated the lactacystin-induced decrease in the level of dopamine (DA) and its metabolites or protected nigral dopaminergic neurons against the lactacystin-induced degeneration. The highest celastrol dose potentiated the lactacystin-induced decrease in the level of DA and its metabolites in the lesioned striatum, and accelerated the lactacystin-induced increase in the oxidative and total metabolism of DA. Moreover, when given alone, this dose of celastrol bilaterally decreased the number and/or density of dopaminergic neurons in the SN. Our results demonstrate that celastrol does not induce neuroprotective effects under conditions of UPS inhibition.

## Introduction

Parkinson’s disease (PD) is a chronic neurodegenerative disorder characterized by degeneration of dopaminergic neurons projecting from the substantia nigra pars compacta (SNc) to the striatum, and the presence of proteinaceous inclusions called Lewy bodies, which are composed predominantly of fibrillar α-synuclein and ubiquitinated proteins (Braak et al. 2004; Ehringer and Hornykiewicz 1960). As a consequence of the loss of striatal dopamine (DA), a progressive impairment of the control of movements occurs, inducing akinesia, rigidity, and resting tremor (Dauer and Przedborski 2003). Although the etiology of PD remains unknown, it is believed to involve numerous risk factors, both genetic and environmental.

The ubiquitin-proteasome system (UPS) is the principal mechanism responsible for the degradation of damaged and misfolded intracellular proteins, and its failure leads to protein accumulation and cell death (Ciechanover and Brundin 2003). A number of studies have suggested that a failure of the UPS may be an important pathogenic factor in PD. In fact, it has been found that mutations of the components related to the UPS, e.g., parkin and ubiquitin carboxy-terminal hydrolase L1 (UCH-L1), lead to degeneration of the nigrostriatal pathway in certain forms of familial PD (Kitada et al. 1998; Leroy et al. 1998). Moreover, an impaired proteasomal function has been described in the SN of idiopathic PD (McNaught and Jenner 2001; McNaught et al. 2003). Besides UPS impairment, cell death in PD has also been linked to neuroinflammatory processes and excessive oxidative stress (Jenner 2003; Qian et al. 2010). In dopaminergic neurons, the oxidative deamination of DA by monoamine oxidase (MAO) and the auto-oxidation of DA results in the production of hydrogen peroxide which in turn can be converted to hydroxyl radicals to react with and cause damage to cellular molecules (Hermida-Ameijeiras et al. 2004).

The involvement of oxidative stress in PD is supported by a postmortem PD brain analysis which involved the evaluation of several parameters, such as protein and DNA oxidation, lipid peroxidation, and decreases in reduced glutathione level (Jenner 1998). The existence of ongoing inflammatory processes that may contribute to the progression of PD is supported, e.g., by the presence of activated microglia, the accumulation of cytokines and nuclear factor kappa B (NF-κB) pathway activation in the cerebrospinal fluid and the brain of PD patients (Hirsch and Hunot 2009; McGeer et al. 1988).

It has been proven that many neuropathological and behavioral features of PD can be replicated in animal models of PD, evoked by the use of UPS inhibitors. For instance, systemic and intracerebral administration of several UPS inhibitors, including lactacystin, epoxomicin, and PSI, induces degeneration of dopaminergic neurons with intracellular inclusions in the SNc as well as behavioral abnormalities in rodents (Fornai et al. 2003; Lorenc-Koci et al. 2011; Mackey et al. 2013; McNaught et al. 2002a, 2004; Vernon et al. 2011; Xie et al. 2010b). The toxicity of UPS inhibitors has also been reported in various cell cultures in vitro (Jantas et al. 2013; McNaught et al. 2002b; Reaney et al. 2006; Rideout et al. 2001). The inhibition of the UPS has been linked with the occurrence of neuroinflammatory processes and oxidative stress. For instance, systemic and intranigral administration of different UPS inhibitors provoke microglial activation in the SN along with the death of dopaminergic neurons (Ahn and Jeon 2006). This is consistent with in vitro data showing microglial activation in cells treated with lactacystin (Kwon et al. 2008). Furthermore, various parameters of oxidative stress have been examined in cell lines treated with UPS inhibitors (Lee et al. 2001). Therefore, it seems that compounds showing antioxidant and anti-inflammatory activity may protect dopaminergic neurons from the UPS failure-induced degeneration.

Celastrol, also called tripterine (3-hydroxy-24-nor-2-oxo-1(10),3,5,7-friedelatetraen-29-oic acid), a quinone methide triterpene, is a pharmacologically active compound extracted from a Chinese herb Tripterygium Wilfordii Hook F (Zhou 1991). Celastrol has strong antioxidant and anti-inflammatory activity and has been found to be effective in a number of animal models of inflammatory (Kiaei et al. 2005; Kim et al. 2009; Li et al. 2008a) and neurodegenerative diseases, e.g., Alzheimer’s and Huntington’s (HD) diseases (Allison et al. 2001; Cleren et al. 2005). Studies have shown that celastrol suppresses microglial activation, pro-inflammatory cytokine production, inducible nitric oxide formation, and lipid peroxidation (Allison et al. 2001; Sassa et al. 1990). Furthermore, celastrol has been reported to possess a potent antitumor activity, both in vitro and in vivo, which is mediated by multiple mechanisms including inhibition of the UPS and induction of apoptosis (Kannaiyan et al. 2011; Yang et al. 2006, 2010).

Recently, it has also been demonstrated that celastrol is able to prevent degeneration of nigrostriatal neurons in the MPTP-induced neurotoxicity in mice and in a genetic *Drosophila* DJ-1A model of PD (Cleren et al. 2005; Faust et al. 2009). In view of the potential antiparkinsonian-like effects of this compound, we decided to test its potency in another PD model, i.e., the lactacystin-induced inhibition of the UPS, which may operate through different pathogenic mechanisms from the above-mentioned models. Therefore, the aim of our study was to determine whether celastrol may exert a neuroprotective effect both in vitro, in the lactacystin-induced toxicity in mouse primary cortical neurons and human neuroblastoma SH-SY5Y cells, and in vivo, in the rat PD model of lactacystin-induced degeneration of nigrostriatal dopaminergic system. Human neuroblastoma SH-SY5Y cell line is widely used to study the mechanism of cell death in relation to PD because it possesses many characteristics of dopaminergic neurons (Påhlman et al. 1990; Xie et al. 2010a). On the other hand, mouse primary cortical neurons exhibit typical neuronal phenotype (Lesuisse and Martin 2002) and we used them to examine the effects of treatment on two types of cells with different features.

## Materials and Methods

### In Vitro Study

#### Chemicals

Dulbecco’s modified Eagle medium (DMEM), fetal bovine serum (FBS), Neurobasal A medium, and supplement B27 were purchased from Gibco (Invitrogen, Poisley, UK). The Cytotoxicity Detection Kit came from Roche Diagnostic (Mannheim, Germany). All the other reagents were from Sigma-Aldrich (Steinheim, Germany).

#### Cell Cultures

##### Mouse Primary Cortical Neurons

**Fig. 1:**
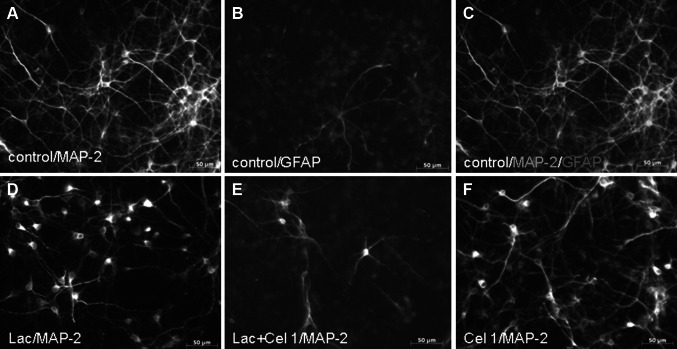
Microphotographs from MAP-2 immunofluorescence of 7DIV mouse primary cortical neurons treated with celastrol (Cel, 1 μM) and lactacystin (Lac, 2.5 μg/ml) for 48 h (**a**, **d**, **e**, and **f**). Celastrol enhanced the reduction in the number of MAP-2 positive cells in lactacystin-treated primary neuronal cell cultures. The purity of neuronal cell cultures (about 90 % neurons) was confirmed by double-immunostainig of vehicle-treated cells with neuronal (anti-MAP-2) and glia (anti-GFAP) specific markers (**a**, **b**, and **c**)

Brain tissues were collected from Swiss mouse embryos on day 15/16 of gestation and were cultured essentially as described previously (Brewer 1995; Jantas-Skotniczna et al. 2006). All the procedures were carried out in accordance with the National Institutes of Health Guidelines for the Care and Use of Laboratory Animals, and were granted an approval from the Bioethics Commission as compliant with the Polish law. The animal care followed the official guidelines, and all efforts were made to minimize the number of animals used and their suffering. Briefly, pregnant females were anesthetized with a CO_2_ vapor, killed by cervical dislocation and subjected to cesarean section in order to dissect fetal brains. To obtain primary cortical neurons, the cortex was dissected from embryonic rat brain. The dissected tissues were separately minced into small pieces, were then digested with trypsin (0.1 %) for 15 min at the room temperature, triturated in the presence of 10 % fetal bovine serum and DNAse I (150 Kunitz units/ml), and finally centrifuged for 5 min at 1,000 rpm. The cells were suspended in Neurobasal medium supplemented with B27 and plated at a density of 1.5 × 10^5^ cells per cm^2^ onto poly-ornithine (0.01 mg/ml)-coated multi-well plates. This procedure typically yields cultures containing >90 % neurons and <10 % supporting cells as verified by immunocytochemistry (Fig. 1). The cultures were then maintained at 37 °C in a humidified atmosphere containing 5 % CO_2_ for 7 days prior to experimentation.

##### Human Neuroblastoma SH-SY5Y Cells

Human neuroblastoma SH-SY5Y cells (ATCC, passages 10–20) were grown in DMEM supplemented with a 10 % heat-inactivated FBS and 0.1 % penicillin/streptomycin mixture. The cells were maintained at 37 °C in a saturated humid atmosphere containing 95 % air and 5 % CO_2._ After reaching 80 % confluency, the cells were subcultured by trypsinization and seeded into multi-well plates with a density of 3 × 10^5^ per ml. The cells were differentiated for 7 days with retinoic acid (RA, 10 μM), added to the culture medium and changed every 3 days. One day before the experiment, the culture medium was replaced with DMEM containing antibiotics and 1 % FBS.

#### Cell Treatment

In the co-treatment mode, primary cortical neurons on day 7 in vitro (7 DIV) were treated with celastrol (0.01, 0.1, 1, 5, and 10 μM) and lactacystin at a concentration of 2.5 μg/ml (6.6 μM) for 48 h. In our previous study, the chosen concentration of lactacystin was shown to evoke *ca*. 40 % reduction in cell viability (Jantas et al. 2011). When primary cortical neurons were pretreated with celastrol for 4 and 18 h before exposure to lactacystin (2.5 μg/ml), only the lower concentrations (0.01, 0.1, and 1 μM) of that compound were used.

RA-differentiated SH-SY5Y cells were incubated with lactacystin (2.5–10 μg/ml) for 48 h in order to choose an effective concentration for study with celastrol. Lactacystin concentration of 10 μg/ml (26.4 μM), which caused *ca*. 40–50 % reduction in cell viability after 48 h, was chosen for the study with celastrol. In the co-treatment mode, celastrol (0.01, 0.1, 1, and 2.5 μM) was added with lactacystin to RA-SH-SY5Y cells for 48 h. In the pretreatment mode, RA-SH-SY5Y cells were treated with the lower concentrations of celastrol (0.01, 0.1, and 1 μM) for 1, 4, and 18 h before exposure to lactacystin (10 μg/ml).

Celastrol stock solution (10 mM) was prepared in DMSO, stored at −20 °C and its fresh dilutions in distilled water were prepared for each experimental set. Lactacystin was dissolved in distilled water. The chemicals were added to the culture medium at indicated concentrations and solvent for celastrol was present in cultures at a final concentration of 0.1 %.

#### Measurement of Lactate Dehydrogenase (LDH) Release

In order to estimate cell death, the level of LDH, released from damaged cells into the culture media, was measured 48 h after the treatment of cells. A colorimetric assay was applied, according to which the amount of formazan salt, formed after the conversion of lactate to pyruvate and then by the reduction of tetrazolium salt, was proportional to LDH activity in a sample. Cell-free culture supernatants were collected from each well and incubated with an appropriate reagent mixture according to the supplier’s instructions (Cytotoxicity Detection Kit, Roche) at the room temperature for 20 min. The intensity of red color formed in the assay and measured at a wavelength of 490 nm, was proportional to the LDH activity and to the number of damaged cells. Data were normalized to the activity of LDH released from vehicle-treated cells (100 %) and were expressed as a percent of the control ± SEM, established from *n* = 5 wells per one experiment from 3 separate experiments.

#### Measurement of Cell Viability (MTT Reduction Assay)

Cell viability assessment was conducted 48 h after the treatment of cells with the agents. Cell damage was quantified using a tetrazolium salt colorimetric assay with 3-[4,5-dimethylthylthiazol-2-yl]-2,5-diphenyltetrazolium bromide (MTT). Briefly, MTT was added to each well (at a final concentration of 0.15 mg/ml) and incubated for 30 min at 37 °C; then the dye was solubilized with DMSO and the absorbance of each sample was measured at 570 nm in a 96-well plate-reader (Multiscan, Labsystem). Data were normalized to the absorbance in the vehicle-treated cells (100 %) and expressed as a percent of the control ± SEM established from *n* = 5 wells per one experiment from 2 to 3 separate experiments.

#### Immunofluorescence

The purity of mouse primary neuronal cell cultures as well the morphological changes in neurons after 48 h of treatment with lactacystin (2.5 μg/ml) and celastrol (1 μM) was determined by immunocytochemistry. Cells were seeded in 24-well plates containing culture glass cover slips covered by poly-ornithine (0.01 mg/ml) at a density of 3 × 10^5^ cells/well. After cell treatment at 7 DIV, the cultures were fixed with 4 % paraformaldehyde, permeabilized with PBS containing 0.25 % Triton X-100 (PBS-TX-100) and blocked with 5 % normal goat serum in PBS-TX-100. Primary antibodies against neuronal (mouse anti-MAP-2, 1:200; Santa Cruz) and glia (rabbit anti-GFAP, 1:400; Sigma) markers were added and incubated with cells for 120 min at RT. After thrice washing in PBS, the cells were incubated for 60 min with secondary antibodies: Alexa Fluor^®^488-labeled goat anti-mouse and Alexa Fluor^®^568-labeled goat anti-rabbit IgG (Invitrogen, USA) diluted 1:500 in PBS. After washing with PBS, cover slips with cells were mounted with ProLong^®^Gold antifade reagent (Invitrogen, USA). Cells were examined using a fluorescence AxioObserver microscope (Carl Zeiss, Germany) equipped with the software Axiovision 3.1 at excitation wavelengths of 470 nm (Alexa Fluor^®^488) and 555 (Alexa Fluor^®^568).

### In Vivo Studies

#### Animals

The study was carried out on male Wistar rats weighing between 310 and 445 g at the beginning of the experiment. The animals were kept under standard laboratory conditions: 5 animals per a large cage, at a room temperature (22 °C) on an artificial light/dark cycle (12/12 h), with free access to standard laboratory food and water. Experiments were carried out according to the National Institutes of Health *Guide for the Care and Use of Laboratory Animals* (publication no. 85–23, revised in 1985) and were approved by the Institute’s Bioethics Commission. All efforts were made to minimize the number of animals used and their suffering.

#### Drugs

Lactacystin and celastrol were provided by the Sigma-Aldrich (Steinheim, Germany).

#### Surgical Procedure

Rats were lightly anesthetized with pentobarbital (Vetbutal, 30 mg/kg i.p. Biowet, Poland) and then were placed in stereotaxic apparatus. A stainless steel cannula (0.28 mm o.d.) was inserted unilaterally through a small hole in the skull and the cannula tip was placed into the left SNc at the following coordinates: 5.3 mm caudal to the bregma, 2 mm laterally on the left side of the sagital suture, and 7.6 mm beneath the skull surface (Paxinos and Watson 1986). Lactacystin, at a single dose of 5 μg in a volume of 2 μl of sterile redistilled water, was slowly injected into the SNc at a flow rate of 0.5 μl/min using a Hamilton microsyringe connected, via polyethylene tubing, to the cannula. The cannula was left in place for an additional 5 min to allow diffusion of the compound away from the cannula tip. Control rats were treated in the same manner but received equivalent volume of vehicle instead of lactacystin. The dose of lactacystin was selected on the basis of our previous research which evaluated the levels of decline in striatal DA after different doses of intranigrally administered lactacystin (Lorenc-Koci et al. 2011). We showed that lactacystin given at a dose of 5 μg/2 μl induced a distinct (over 80 %) decrease in the striatal DA level 1 week after intranigral administration. After surgery, animals were returned to their home cages.

#### Treatment

Eight groups of rats were used for the study. Lactacystin- and vehicle-injected rats were treated systemically with celastrol (0.3, 1 or 3 mg/kg/1 ml i.p.) or vehicle (1 ml/kg) for 4 days (1 day before surgery and then for 3 consecutive days). Celastrol was dissolved in a mixture of dimethylsulfoxide (DMSO) and propylene glycol (1:9, v/v). A stock solution of celastrol was prepared in DMSO, stored in small aliquots at −20 °C and freshly diluted before use. The dose of celastrol was chosen on the basis of other rodent studies (Cleren et al. 2005; Yang et al. 2006).

#### High Performance Liquid Chromatography (HPLC) Procedure

One week after surgery, the rats were killed by decapitation and their left and right striata were rapidly dissected on an ice-chilled plate and frozen at −80 °C until an HPLC analysis. Tissue samples were weighted and homogenized in an ice-cold 0.1 M perchloric acid containing 0.05 mM ascorbic acid. After sonification, the homogenates were centrifuged at 12,000 rpm for 15 min at 4 °C, and the supernatants were filtered through 0.2-μm cellulose filters (Alltech Associates Inc.; Deerfield, IL, USA). The supernatants were used to determine the concentrations of DA and its metabolites homovanillic acid (HVA), 3,4-dihydroxyphenylacetic acid (DOPAC) and 3-methoxytyramine (3-MT) separately for the right and the left side using the HPLC system equipped with a C18 column (Thermo Fisher Scientific Inc., Waltham, MA, USA) and a Coulochem III detector (ESA Inc.; Chelmsford, MA, USA). The mobile phase consisted of 50 mM citrate–phosphate buffer (pH 4.2), 0.25 mM EDTA, 0.25 mM sodium octyl sulfonate, 2.4 % methanol, and a 1.3 % acetonitrile. The column temperature was set at 32 °C and the flow rate was maintained at 0.8 ml/min. The levels of DA and its metabolites in test samples were quantified by comparisons of the peak area with standards that were run on the day of analysis. Data were collected and analyzed using Chromeleon 6.8 software. Values were expressed as ng/g wet weight of the tissue.

#### Immunohistochemistry

##### Tissue Preparation

Posterior parts of the brain containing the whole SN were fixed in a buffered 4 % paraformaldehyde for about 20 h at 4 °C and were then immersed (at 4 °C) in a buffered 20 % sucrose for cryoprotection. After a few days, the brains were cut up on a freezing microtome into 30-μm frontal sections. Every sixth serial section within the entire length of the SN was sampled. Free-floating sections were incubated for 48 h at 4 °C in a mouse monoclonal anti-tyrosine hydroxylase (TH) antibody (Chemicon AB), diluted at 1:3,000, and then rinsed in a phosphate-buffered saline and processed by an avidin–biotin-peroxidase complex method using an ABC-peroxidase kit (Vector Laboratory) and diaminobenzidine as a chromogen. The stained sections were mounted onto slides, dried, dehydrated, cleared in xylene, and cover-slipped in a Canada balsam.

##### Stereological Counting

TH-immunoreactive (TH-ir) neurons in the whole SN (pars compacta + reticulata) of rats were stereologically counted on both sides of the brain using a microscope (Leica, DMLB; Leica, Denmark) equipped with a projecting camera and a microscope stage connected to a xyz stepper (PRIOR ProScan) controlled by the computer using the Visiopharm New CAST software as described previously (Lorenc-Koci et al. 2011). Systemic, uniform, and random sampling were used to choose sections for the analysis. For stereological estimation, cell counts were performed within the contours of the SN [AP = −4.80 to −6.30 mm from the bregma according to the atlas by Paxinos and Watson (1986)] in at least 8–10 sections at 180 mm intervals. The SN was outlined under a lower magnification (5×), and uniformly sampled dissector points were randomly used along the whole structure using the meander sampling. The total number of TH-ir neurons in the SN was unbiasedly estimated using a three-dimensional probe under a higher magnification (63×) according to the formula: *N* = ΣQ × *V*(ref)/*ν*(dis) × Σ*P*, where ΣQ is the total count of TH-ir neurons in uniformly sampled dissectors, *V*(ref)—the total volume of the SN, *v*(dis)—the total volume of the dissector (Sterio 1984), and Σ*P*—the total number of all dissector points. A counting frame of 57.63 × 57.63 µm (3,321.2 μm^2^), a sampling grid of 257.33 × 257.33 µm (66,218.7 μm^2^), and a dissector height of 15 µm below the surface were employed. Sampling was optimized to produce a coefficient of error well under the observed biological variability (Gundersen et al. 1999). The total volume of the SN *V*(ref) was estimated using Cavalieri’s principle (Gundersen et al. 1999) according to the formula: *V*(ref) = *t* × *a*(*p*) × ΣP, where t is the known distance between sections, *a*(*p*) is the area associated with each point of a grid, and ΣP is the total number of the counted points over all sections from one rat.

#### Data Analysis

All statistical analyses were performed using the Statistica software package (StatSoft Inc.; Tulsa, USA). Body weight was expressed for each rat as the percent of the initial value calculated as the mean value of the individual groups. Body weight changes were assessed using a two-way analysis of variance (ANOVA) for repeated measures with a split-plot design (treatment as a between-subject factor, and time as a within-subject factor), followed by the Neuman-Keuls test for a post-hoc comparison. The HPLC-derived data and immunohistochemical data were analyzed by a two-way ANOVA, followed by Neuman-Keuls test. Data from in vitro studies after normalization as a percentage of the control ± SEM were analyzed using a one-way ANOVA and the post-hoc Tukey test for multiple comparisons. All the hypotheses were tested at a significance level of 0.05.

## Results

### In Vitro Studies

#### Mouse Primary Cortical Neurons

**Fig. 2:**
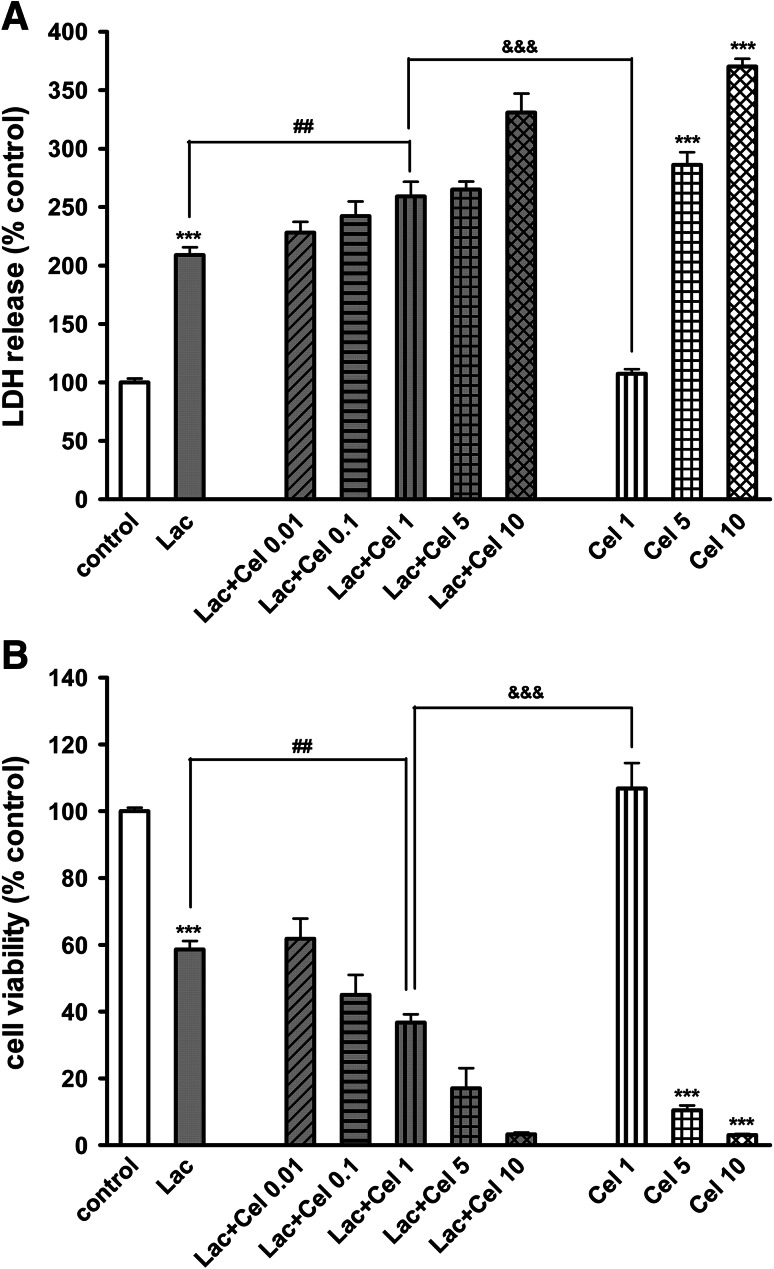
The effect of celastrol (Cel; 0.01–10 μM) on the lactacystin (Lac; 2.5 μg/ml)-induced LDH release (**a**) and the lactacystin-evoked reduction in cell viability (**b**) in 7DIV primary cortical neurons. The cells were co-treated with lactacystin and celastrol for 48 h. The data were normalized and calculated as a percentage of the control value and they represent the mean ± SEM from 3 separate experiments (*n* = 15). ***p < 0.001 versus vehicle-treated cells; ^##^ *p* < 0.01 versus Lac-treated cells; ^&&&^ *p* < 0.001 versus Cel (1 μM)-treated cells

Lactacystin (2.5 μg/ml) evoked about 50 % increase in LDH release and 40 % reduction of cell viability after 48-h treatment of 7 DIV cortical neurons (Fig. 2a, b). Celastrol (5 and 10 μM) given alone in a concentration-dependent way induced cell death after 48-h treatment. Concomitant administration of a subtoxic concentration of celastrol (1 μM) and lactacystin (2.5 μg/ml) evoked a statistically significant increase in cell death, measured by the LDH and MTT assays compared to cells treated with a single agent (Fig. 2a, b). No additive effect was observed when higher concentrations of celastrol (5 and 10 μM) were used. The biochemical data on subtoxic celastrol (1 μM) increasing the lactacystin-induced cell damage were confirmed by morphological analysis of anti-MAP-2 immunostained neurons (Fig. 1).

Pretreatment of neuronal cell cultures for 4 and 18 h with celastrol (0.01 an 0.1 μM) before lactacystin (2.5 μg/ml) did not evoke any significant changes in cell viability measured by the MTT assay when compared to the lactacystin-treated cells (Table 1). However, like in the concomitant exposure paradigm, subtoxic celastrol (1 μM) given 4 and 18 h before lactacystin, significantly increased the cell damage induced by lactacystin (Table 1).

**Table 1:** The effect of celastrol pretreatment on lactacystin-induced cell damage in primary cortical neurons

Groups	Cell viability (% control)	
	Pre-4 h	Pre-18 h
Control	100.00 ± 2.92	99.99 ± 2.82
Cel 1	105.18 ± 2.16	99.74 ± 3.52
Lac	57.11 ± 3.44***	57.32 ± 1.98***
Lac + Cel 0.01	65.81 ± 3.52	62.39 ± 1.72
Lac + Cel 0.1	62.02 ± 2.25	67.48 ± 3.66
Lac + Cel 1	23.84 ± 0.45^###,&&&^	39.96 ± 1.86^###,&&&^

The 7 DIV neuronal cell cultures were pretreated with celastrol (Cel; 0.01; 0.1 and 1 μM) for 4 and 18 h before lactacystin (Lac, 2.5 μg/ml) administration. After 48 h incubation of cells with lactacystin, the cell viability was estimated by MTT reduction assay. The data were normalized and calculated as a percentage of the control value and they represent the mean ± SEM from 2 separate experiments (*n* = 10)

*** *p* < 0.001 versus vehicle-treated cells; ^###^ *p* < 0.001 versus Lac-treated cells; ^&&&^ *p* < 0.001 versus Cel (1 μM)-treated cells

#### Human Neuroblastoma RA-SHSY5Y Cells

**Fig. 3:**
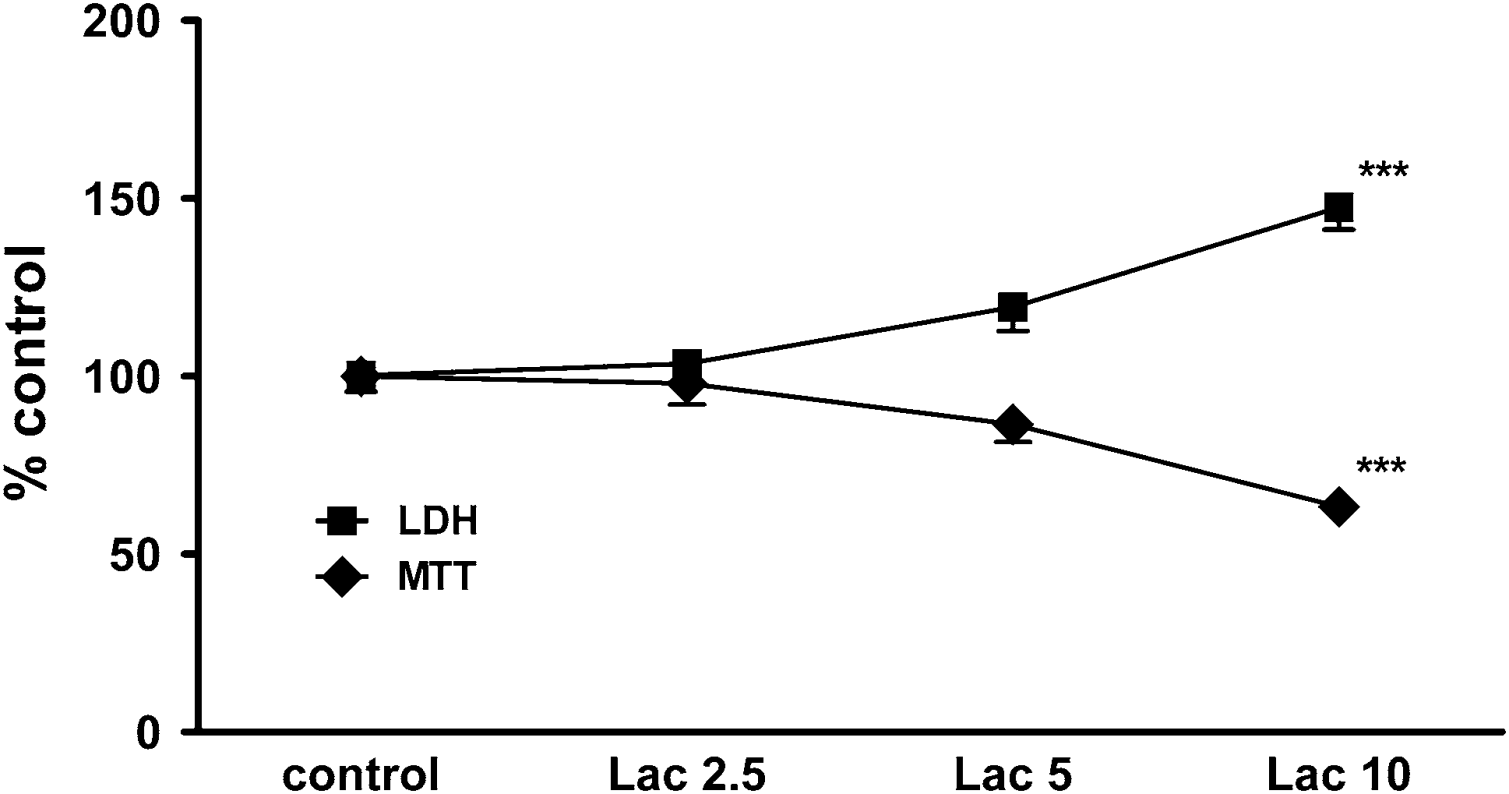
The time-course of the effect of lactacystin (Lac; 2.5–10 μg/ml) on the LDH release and cell viability in retinoic acid (RA)-differentiated SH-SY5Y cells. The cells were treated with lactacystin for 48 h. The data were normalized and calculated as a percentage of the control value and they represent the mean ± SEM from 3 separate experiments (*n* = 15). ***p < 0.001 versus vehicle-treated cells

**Fig. 4:**
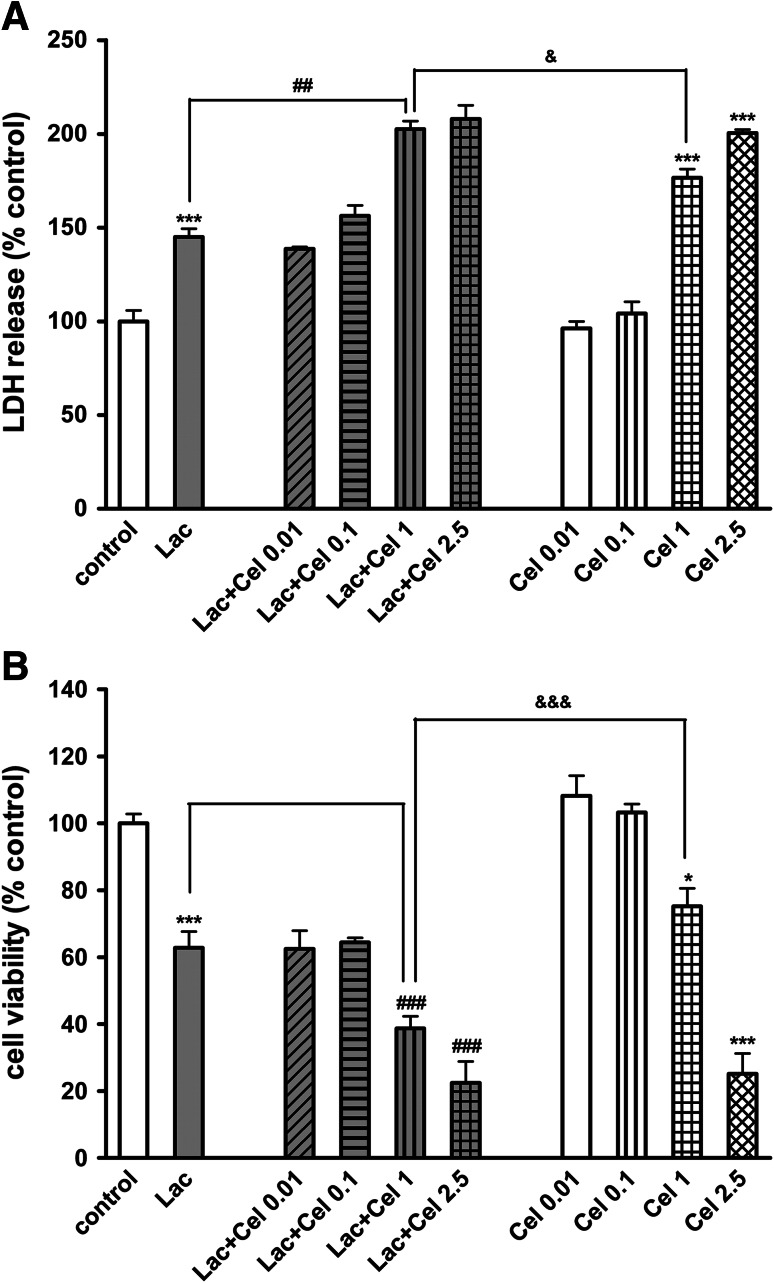
The effect of celastrol (Cel; 0.01–2.5 μM) on the lactacystin (Lac; 10 μg/ml)-induced LDH release (**a**) and the lactacystin-evoked reduction in cell viability (**b**) in RA-SH-SY5Y cells. The cells were co-treated with lactacystin and celastrol for 48 h. The data were normalized and calculated as a percentage of the control value and they represent the mean ± SEM from 3 separate experiments (*n* = 15). *p < 0.05 and ***p < 0.001 versus vehicle-treated cells; ^##^ *p* < 0.01 and ^###^ *p* < 0.001 versus Lac-treated cells; ^&^ *p* < 0.05 and ^&&&^ *p* < 0.001 versus Cel (1 μM)-treated cells

Lactacystin (10 μg/ml) evoked a significant increase in LDH release (*ca*. 50 %) and *ca*. 40 % reduction of cell viability after 48-h treatment (Fig. 3). Celastrol (1 and 2.5 μM) given alone in a concentration-dependent manner induced cell death in RA-SHSY5Y cells (Fig. 4a, b). A significant increase in cell death was observed after concomitant treatment of cells with a toxic concentration of celastrol (1 μM) and lactacystin (10 μg/ml) compared to cells treated with one agent only (Fig. 4a, b).

Pretreatment of RA-SH-SY cells for 1, 4, and 18 h with celastrol (0.01 an 0.1 μM) before lactacystin (10 μg/ml exposure) did not evoke any significant changes in cell viability/toxicity parameters measured by MTT and LDH assays (Table 2). However, like in the concomitant exposure paradigm, celastrol (1 μM) given 1, 4, and 18 h before lactacystin significantly increased cell damage when compared to cells treated only with lactacystin or celastrol (Table 2).

**Table 2:** The effect of celastrol pretreatment on lactacystin-induced cell damage in RA-SH-SY5Y cells

Groups	Cell viability (% control)			LDH release (% total)		
	Pre-1 h	Pre-4 h	Pre-18 h	Pre-1 h	Pre-4 h	Pre-18 h
Control	100.00 ± 6.58	100.00 ± 4.21	102.40 ± 1.10	15.36 ± 1.76	16.80 ± 1.27	12.17 ± 2.68
Cel 1	76.07 ± 3.72^***^	70.70 ± 3.28^***^	73.73 ± 6.49^***^	28.81 ± 1.91^***^	33.61 ± 1.69^***^	27.67 ± 3.57^*^
Lac	57.84 ± 1.09^***^	57.62 ± 1.72^***^	46.47 ± 1.09^***^	46.87 ± 1.58^***^	43.55 ± 1.58^***^	38.34 ± 1.4^***^
Lac + Cel 0.01	58.40 ± 2.44	59.91 ± 2.54	53.74 ± 2.88	45.06 ± 1.83	40.86 ± 1.68	39.10 ± 2.39
Lac + Cel 0.1	55.61 ± 2.58	50.85 ± 0.76	50.40 ± 3.85	44.43 ± 1.96	41.46 ± 1.35	42.06 ± 3.38
Lac + Cel 1	42.41 ± 1.60^## &&^	34.97 ± 1.61^### &&^	29.59 ± 2.08^###^^&&&^	62.22 ± 2.62^### &&&^	63.08 ± 2.43^### &&&^	69.36 ± 3.99^### &&&^

The cells were pretreated with celastrol (Cel; 0.01; 0.1 and 1 μM) for 1, 4, and 18 h before lactacystin (Lac, 10 μg/ml) administration. After 48 h incubation of cells with lactacystin, the cell viability and cell toxicity were estimated by the MTT reduction test and the LDH release assay, respectively. The data w ere normalized and calculated as a percentage of the control value (MTT assay) or as a percentage of total cell damage (cells treated with 1 % TritonX100) and are presented as the mean ± SEM from 2 separate experiments (*n* = 10)

*** *p* < 0.001 versus vehicle-treated cells; ^#^ *p* < 0.05, ^##^ *p* < 0.01 and ^###^ *p* < 0.001 versus Lac-treated cells; ^&&^ *p* < 0.01 and ^&&&^ *p* < 0.001 versus Cel (1 μM)-treated cells

### In Vivo Studies

#### Body Weight Changes

**Fig. 5:**
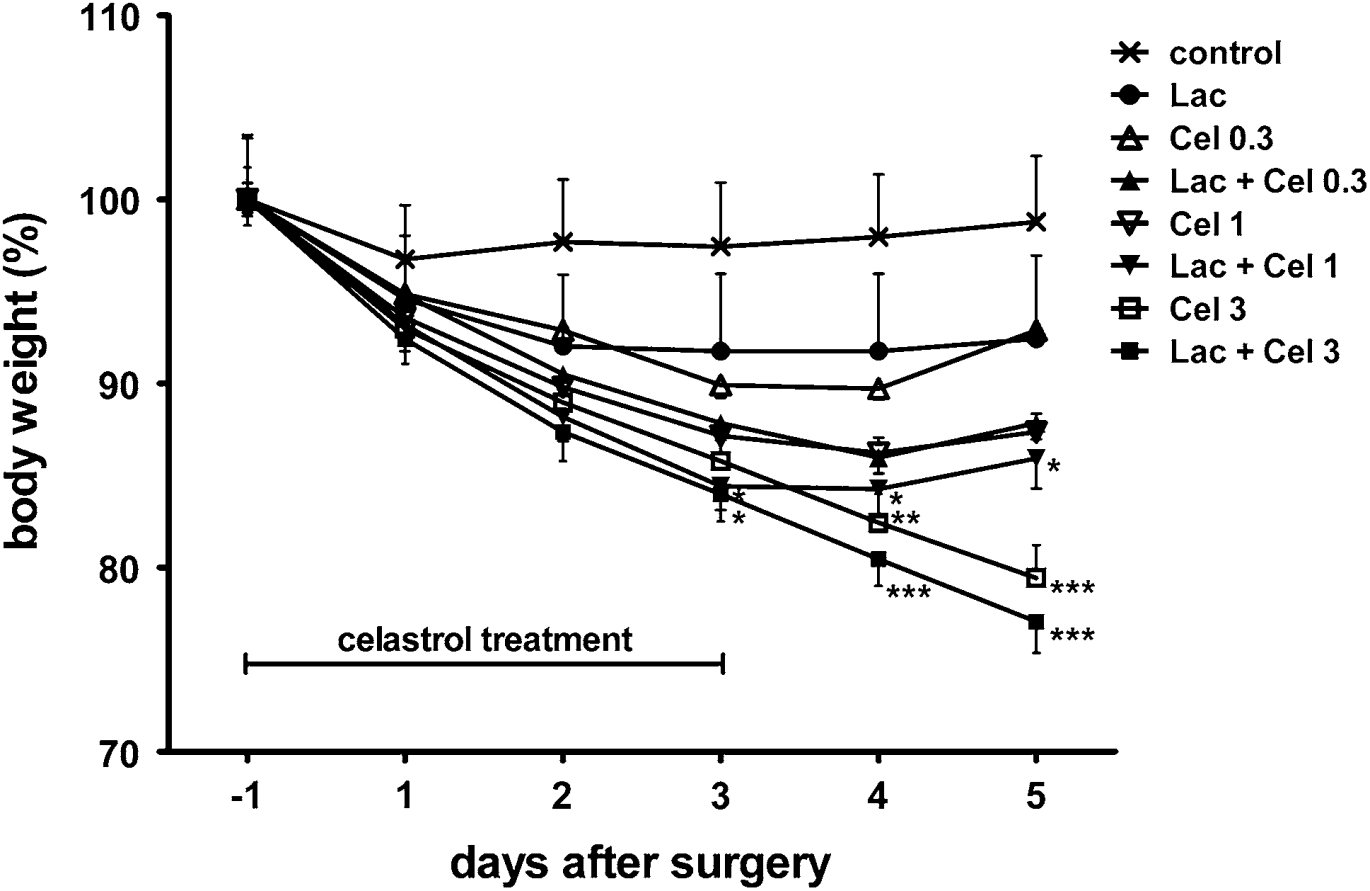
The effect of lactacystin (Lac) and celastrol (Cel) on the rat body weight. Lactacystin (5 μg/2 μl) was administered unilaterally into the left SNc. Celastrol (0.3, 1 or 3 mg/kg i.p) was given subchronically for 4 days (1 day before surgery and then for three consecutive days). The number of animals per group: 8–10. The data were calculated as a percentage of the control value and they are expressed as the mean ± SEM. The symbols indicate significant differences in the post-hoc test: *p < 0.05, **p < 0.01, ***p < 0.001 versus control

General health of the animals was checked daily. Body weight analysis was performed up to 5 days after the surgery. A 2-way ANOVA for repeated measures showed a significant main effect of celastrol (*F*_3,65_ = 7.12, *p* < 0.001) and time (*F*_5,325_ = 615.05, *p* < 0.001), and significant interactions: celastrol × time (*F*_15,325_ = 52.51, *p* < 0.001) and lactacystin × time (*F*_5,325_ = 15.10, *p* < 0.001). A post-hoc analysis showed the significant decrease in body weight on the fourth day after surgery in non-lesioned rats treated with the highest dose of celastrol (3 mg/kg) (Fig. 5). In the lesioned animals, the significant decrease in body weight was observed on the third day after surgery in groups treated with 1 and 3 mg/kg of celastrol. However, only the highest celastrol dose (3 mg/kg) induced an irreversible body weight loss in both lesioned and non-lesioned animals, even after celastrol withdrawal and some of these rats (27 %) died before the end of the experiment. No mortality was reported in other groups of animals. Groups treated with lower doses of celastrol (0.3 or 1 mg/kg) ceased to lose weight on the second day after celastrol withdrawal. On the seventh day after surgery, body weight in these two groups was similar and was approximately 90 % of the initial value (data not shown).

#### Striatal DA Metabolism

**Fig. 6:**
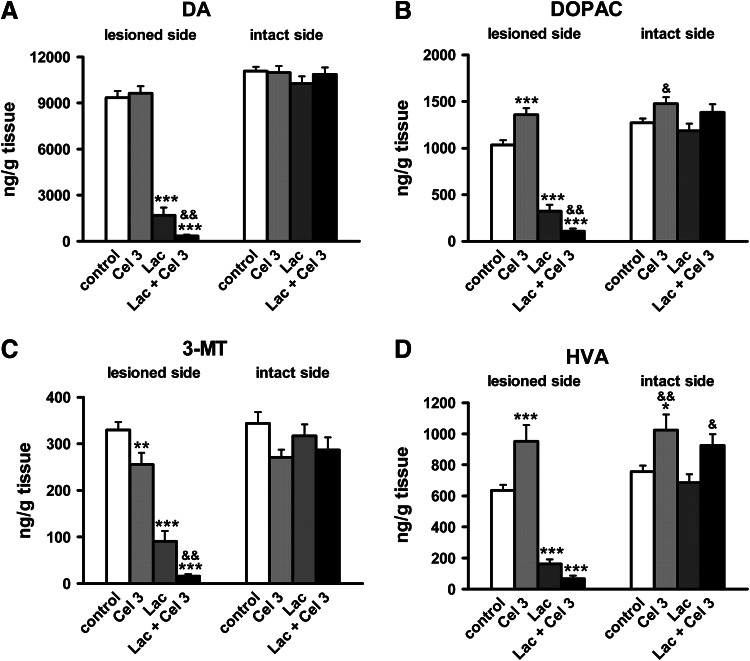
The effect of unilateral administration of lactacystin (Lac; 5 μg/2 μl) into the left SNc and intraperitoneal administration of celastrol (Cel; 3 mg/kg) on the level of DA (**a**) and its metabolites DOPAC (**b**) 3-MT (**c**) and HVA (**d**) in the striatum. The rats were killed 7 days after lactacystin administration. Lactacystin induced a strong decrease in the striatal levels of DA and its metabolites, which was potentiated by celastrol. The number of animals per group: 8–10. The values are expressed as the mean ± SEM. The *symbols* indicate significant differences in the post-hoc test: *p < 0.05, **p < 0.01, ***p < 0.001 versus control group; ^&^ *p* < 0.05, ^&&^ *p* < 0.01 versus Lac group, on the same side of the striatum

One week after lesion, the rat striata were examined for DA, DOPAC, 3-MT, and HVA content by HPLC. A two-way ANOVA was used to analyze the main effect of lactacystin and the highest dose of celastrol (3 mg/kg), separately for the ipsilateral (lesioned) and contralateral (intact) side of the striatum (Fig. 6a–d). In the ipsilateral striatum, the main significant effects of lactacystin were observed for DA, DOPAC, 3-MT, and HVA levels (*F*_1,33_ = 446.51, *p* < 0.001; *F*_1,33_ = 301.42, *p* < 0.001; *F*_1,33_ = 175.20, *p* < 0.001; *F*_1,33_ = 169.65, *p* < 0.001, respectively), whereas the main effect of celastrol was apparent for 3-MT and HVA levels (*F*_1,33_ = 16.86, *p* < 0.001; *F*_1,33_ = 4.51.86, *p* < 0.05, respectively). There were also significant interactions between lactacystin and celastrol for DA, DOPAC, and HVA levels (*F*_1,33_ = 4.29, *p* < 0.05; *F*_1,33_ = 23.64, *p* < 0.001; *F*_1,33_ = 15.55, *p* < 0.001, respectively). A Newman–Keuls post-hoc test showed no decrease in the level of DA in the ipsilateral striatum of rats treated with celastrol alone compared to the ipsilateral striatum of the control animals (Fig. 6a); on the other hand, there was an increase in DOPAC and HVA levels (Fig. 6b, d), and a decrease in 3-MT level (Fig. 6c). In contrast, lactacystin induced a dramatic decrease in the levels of striatal DA and its metabolites DOPAC, 3-MT, and HVA compared to the control group (Fig. 6a–d). Combined treatment with lactacystin and celastrol induced a further decrease in the striatal DA, DOPAC, and 3-MT levels, and showed a tendency to decrease HVA level compared to the lactacystin group (Fig. 6a–d).

**Fig. 7:**
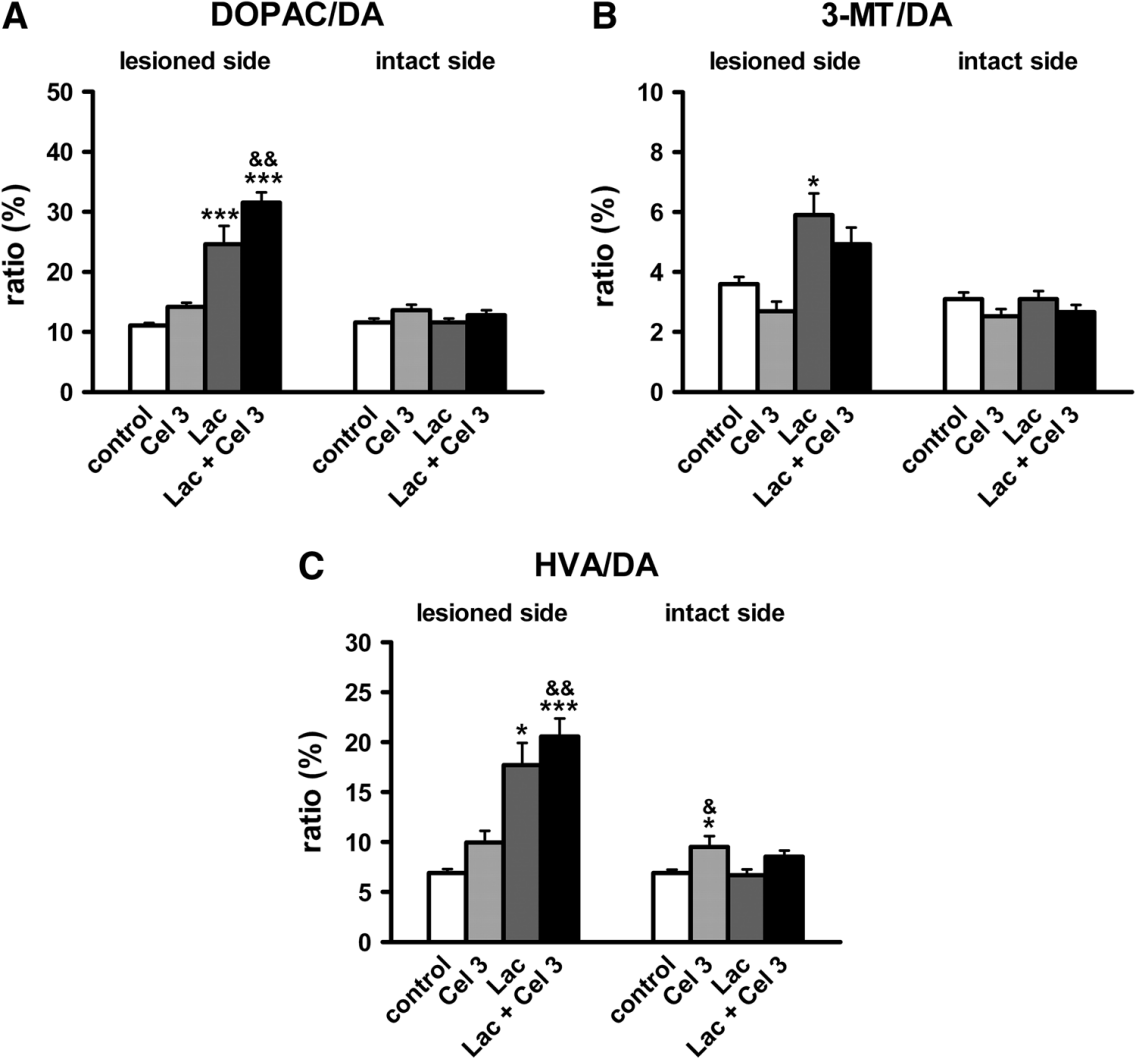
The effect of unilateral administration of lactacystin (Lac; 5 μg/2 μl) into the left SNc and intraperitoneal administration of celastrol (Cel; 3 mg/kg) on DA turnover in the striatum. MAO-dependent oxidative DA deamination (**a**), COMT-dependent *O*-methylation (**b**), and total DA catabolism (**c**), are expressed as a percent of DOPAC/DA, 3-MT/DA, and HVA/DA, respectively. Lactacystin induced an increase in all the metabolic ratios measured. Combined treatment with lactacystin and celastrol potentiated the lactacystin effect on the DOPAC/DA and HVA/DA ratios. The number of animals per group: 8–10. The values are expressed as the mean ± SEM. The values are expressed as the mean ± SEM. *Symbols* indicate significant differences in the post-hoc test: * *p* < 0.05, ***p < 0.001 versus control group; ^&^ *p* < 0.05, ^&&^ *p* < 0.01 versus Lac group^,^ on the same side of the striatum

As regards DA catabolism (Fig. 7a–c) in the ipsilateral striatum, the main effects of lactacystin were observed for MAO-dependent oxidative DA deamination (DOPAC/DA), COMT-dependent *O*-methylation (3-MT/DA), and total DA catabolism (HVA/DA) (*F*_1,33_ = 73.75, *p* < 0.001; *F*_1,33_ = 20.91, *p* < 0.001; *F*_1,33_ = 31.23, *p* < 0.001, respectively), and the main effects of celastrol for the DOPAC/DA and HVA/DA ratios (*F*_1,33_ = 7.82, *p* < 0.01; *F*_1,33_ = 10.16, *p* < 0.01, respectively); however, there was no significant interaction between lactacystin and celastrol (*p* > 0.05). The Newman-Keuls post-hoc test revealed that celastrol alone did not change, whereas lactacystin significantly increased, the striatal metabolic ratios of DOPAC/DA, 3-MT/DA and HVA/DA in the ipsilateral striatum compared to the control group (Fig. 7a–c). Combined treatment with lactacystin and celastrol induced a further increase in the metabolic ratios of DOPAC/DA and HVA/DA compared to the lactacystin group (Fig. 7a, c).

In the contralateral striatum, the only significant main effect of celastrol (3 mg/kg) was observed for DOPAC, 3-MT, and HVA as well for the DOPAC/DA, 3-MT/DA, and HVA/DA ratios (*F*_1,33_ = 7.98, *p* < 0.01; *F*_1,33_ = 4.51, *p* < 0.05; *F*_1,33_ = 14.19, *p* < 0.001; *F*_1,33_ = 4.93, *p* < 0.05; *F*_1,33_ = 4.63, *p* < 0.05; *F*_1,33_ = 11.33, *p* < 0.01, respectively). A post-hoc analysis showed that celastrol significantly increased DOPAC and HVA levels (Fig. 7b, d) as well as the HVA/DA ratio (Fig. 7c) compared to the contralateral striatum of the control group and lactacystin group (rats which received lactacystin on the opposite side of the SN).

The effects of the lower doses of celastrol (0.3 and 1 mg/kg) were presented in Table 3. In the ipsilateral striatum, a two-way ANOVA revealed the main significant effects of lactacystin for DA, DOPAC, 3-MT, and HVA levels (*F*_1,49_ = 433.85, *p* < 0.001; *F*_1,49_ = 319.28, *p* < 0.001; *F*_1,49_ = 307.67, *p* < 0.001; *F*_1,49_ = 408.89, *p* < 0.001, respectively), whereas the main effect of celastrol was apparent for DA, DOPAC, and HVA levels (*F*_2,49_ = 3.71, *p* < 0.05; *F*_2,49_ = 8.49, *p* < 0.001; *F*_2,49_ = 4.55, *p* < 0.05, respectively). Generally, there was no difference in the effects of two lower doses of celastrol on DA metabolism. In contrast to the highest dose of celastrol, there was no significant interaction between lactacystin and any of two lower doses of celastrol (*p* > 0.05). Unlike the sharp decreases in the levels of striatal DA and its three metabolites after lactacystin treatment, as revealed by the Newman-Keuls post-hoc test, only a moderate, but significant decrease in DOPAC level was observed after celastrol (0.3 and 1 mg/kg).

**Table 3:** The effect of unilateral administration of lactacystin (Lac; 5 μg/2 μl) into the left SNc and intraperitoneal administration of lower doses of celastrol (Cel; 0.3 and 1 mg/kg) on striatal level of DA and its metabolites, and striatal DA catabolism

	Groups					
	Control	Cel 0.3	Cel 1	Lac	Lac + Cel 0.3	Lac + Cel 1
Lesioned striatum						
DA	9,332.9 ± 454.82	8,699.4 ± 649.85	7,942.1 ± 660.62	1,695.6 ± 490.97 ***	653.0 ± 131.41***	735.5 ± 208.09***
DOPAC	1,033.7 ± 51.99	888.4 ± 45.67*	827.0 ± 46.44*	324.2 ± 67.51***	151.3 ± 23.69*** ^&^	174.7 ± 42.57*** ^&^
3-MT	329.8 ± 17.03	342.4 ± 16.86	283.5 ± 16.66	90.5 ± 22.35***	56.9 ± 8.63***	71.6 ± 18.41***
HVA	636.1 ± 34.92	541.3 ± 23.60	624.1 ± 50.00	162.3 ± 30.26***	77.8 ± 12.16***	80.8 ± 20.34***
DOPAC/DA	11.1 ± 0.43	10.4 ± 0.46	10.6 ± 0.46	24.6 ± 3.03***	25.3 ± 1.44***	26.2 ± 1.02***
3-MT/DA	3.6 ± 0.24	4.0 ± 0.26	3.7 ± 0.20	5.9 ± 0.72*	9.8 ± 0.81*** ^&&&^	10.3 ± 0.38*** ^&&&^
HVA/DA	6.9 ± 0.39	6.4 ± 0.38	8.0 ± 0.46	13.7 ± 2.21**	13.1 ± 1.25**	11.8 ± 0.99*
Intact striatum						
DA	11,077.4 ± 280.08	12,130.6 ± 285.69	11,185.6 ± 295.35	10,267.0 ± 464.88	11,984.5 ± 334.91	11,075.1 ± 333.15
DOPAC	1,272.9 ± 46.36	1,134.8 ± 31.68	1,104.3 ± 44.78	1,187.8 ± 74.97	1,086.2 ± 29.61	1,093.4 ± 41.40
3-MT	343.9 ± 24.13	351.7 ± 14.49	308.6 ± 14.11	316.8 ± 24.96	311.6 ± 10.35	345.1 ± 9.41
HVA	756.9 ± 38.56	706.5 ± 41.85	812.4 ± 57.96	685.2 ± 54.04	772.1 ± 39.09	720.4 ± 41.44
DOPAC/DA	11.6 ± 0.62	9.4 ± 0.11**	9.9 ± 0.26*	11.6 ± 0.64	9.1 ± 0.26** ^&&^	9.9 ± 0.20** ^&^
3-MT/DA	3.1 ± 0.22	2.9 ± 0.14	2.8 ± 0.14	3.1 ± 0.26	2.6 ± 0.13	3.1 ± 0.11
HVA/DA	6.9 ± 0.34	5.8 ± 0.28	7.2 ± 0.38	6.7 ± 0.56	6.5 ± 0.35	6.5 ± 0.30

The number of animals per group: 8–10. The values are expressed as the mean ± SEM. The symbols indicate significant differences in the post-hoc test: * *p* < 0.05, ** *p* < 0.01, *** *p* < 0.001 versus control group; ^&^ *p* < 0.05, ^&&^ *p* < 0.01, ^&&&^ *p* < 0.001 versus Lac group, on the same side of the striatum

As regards DA catabolism in the ipsilateral striatum, the main effects of lactacystin were observed for all three catabolic ratios: DOPAC/DA, 3-MT/DA, and HVA/DA (*F*_1,49_ = 145.73, *p* < 0.001; *F*_1,49_ = 132.90, *p* < 0.001; *F*_1,49_ = 36.26, *p* < 0.001, respectively), and the main effects of celastrol were seen only for the 3-MT/DA ratio (*F*_2,49_ = 11.85, *p* < 0.001). There was also a significant interaction between lactacystin and celastrol for the 3-MT/DA ratio (*F*_2,49_ = 9.68, *p* < 0.001). In the post-hoc analysis, similarly to the highest celastrol dose, there was no effect of the lower doses of celastrol on any of the catabolic ratios. In contrast, there was no further increase in the DOPAC/DA or HVA/DA ratios after combined treatment, but there was a significant increase in the 3-MT/DA ratio.

In the contralateral striatum, the only significant main effect of celastrol was observed for the DOPAC level, as well for the DOPAC/DA ratio (*F*_2,49_ = 4.81, *p* < 0.05; *F*_2,49_ = 17.64, *p* < 0.001, respectively). A post-hoc analysis showed that two doses of celastrol slightly but significantly decreased the DOPAC/DA ratio, as compared to the control and lactacystin group.

#### Stereological Analysis of the SN

**Fig. 8:**
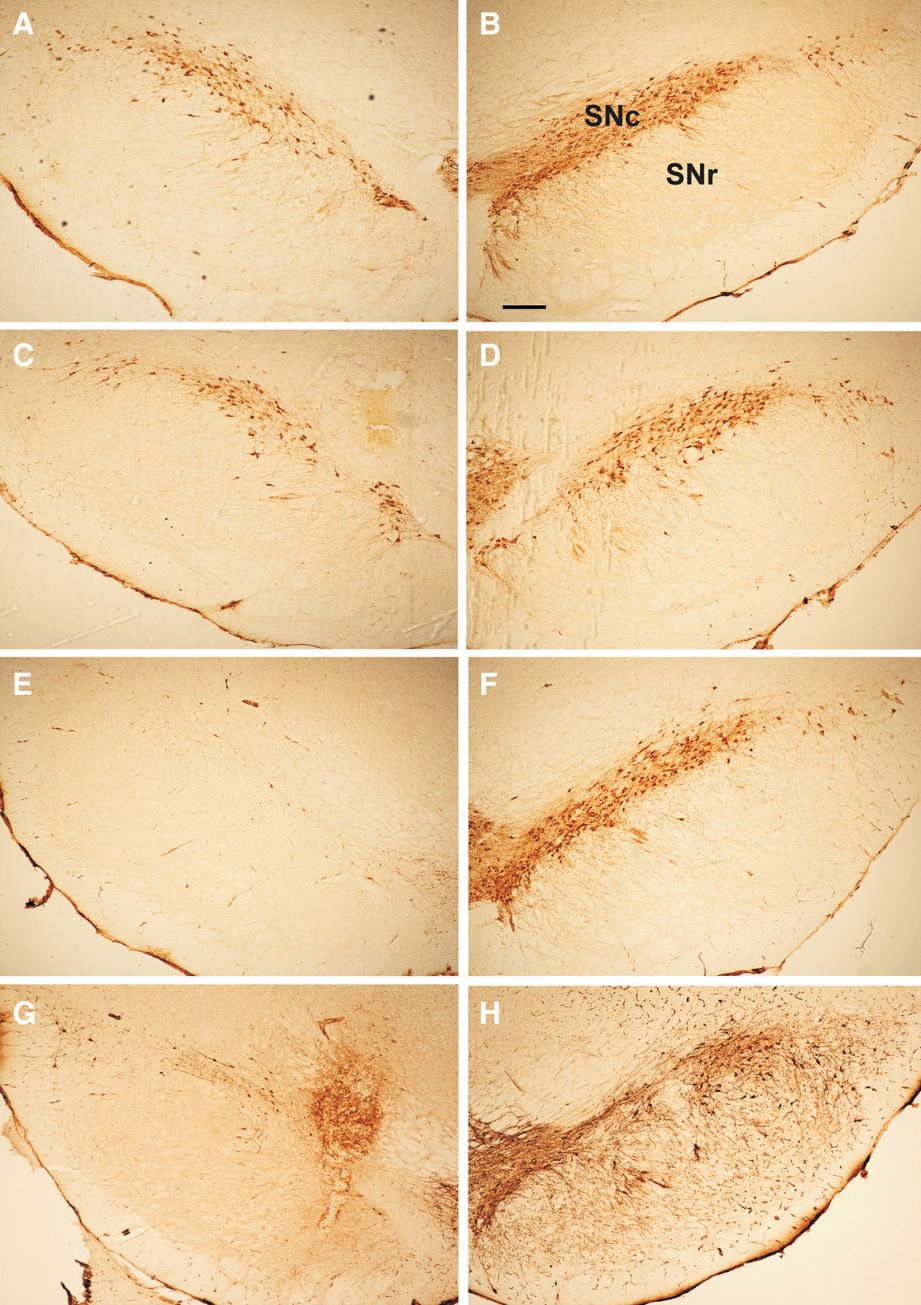
Representative photomicrographs of coronal sections of the rat SN showing the distribution of TH-ir neurons ipsilateral (**a**, **c**, **e**, **g**) and contralateral (**b**, **d**, **f**, **h**) to the injection site. **a**, **b** Control rats; **c**, **d** rats after 4-day celastrol (3 mg/kg) treatment; **e**, **f** rats after unilateral lactacystin (5 μg/2 μl) injection into the SNc; **g**, **h** rats after lactacystin (5 μg/2 μl) administration into the SNc and 4-day celastrol (3 mg/kg) treatment. Calibrations bars = 200 μm. (*SNc* substantia nigra pars compacta, *SNr* substantia nigra pars reticulata)

**Fig. 9:**
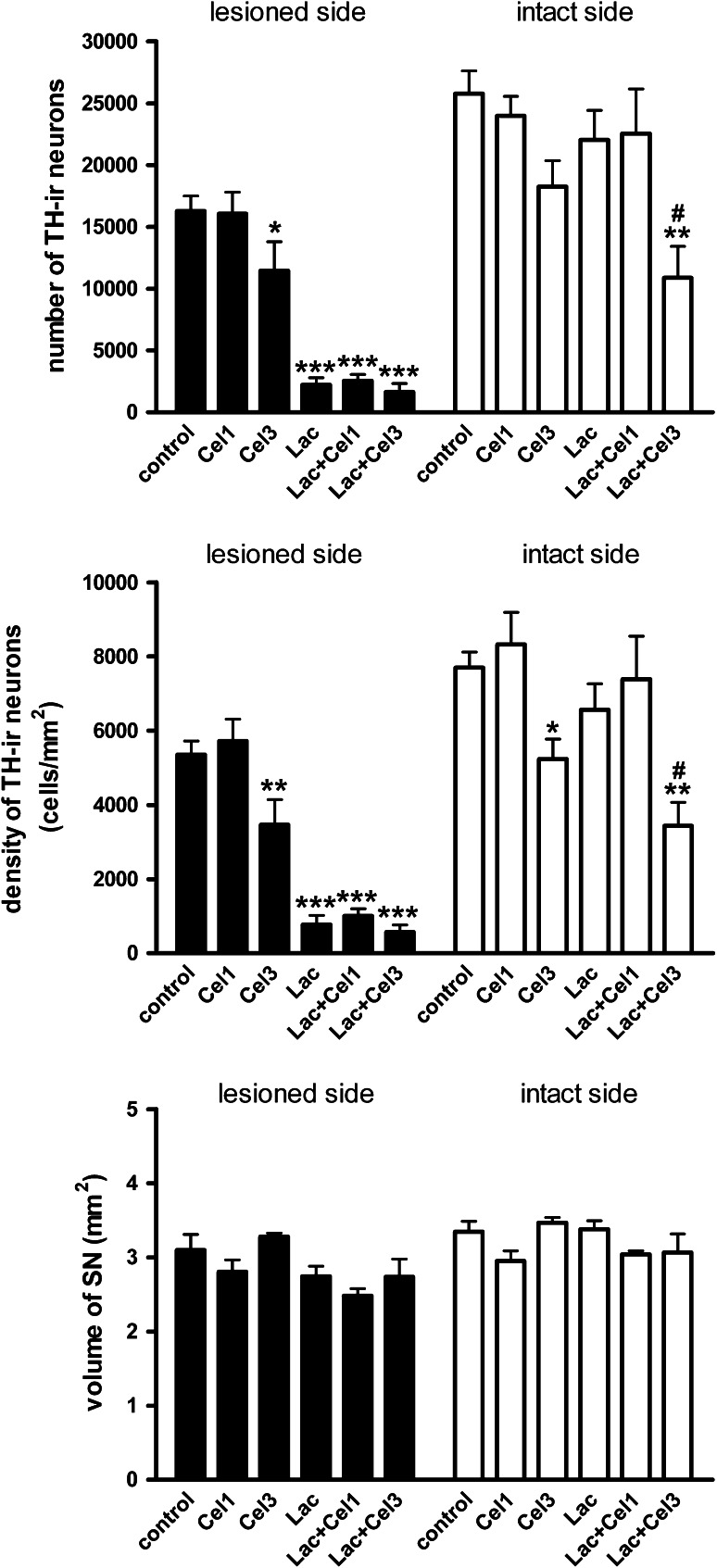
The effects of unilateral administration of lactacystin (Lac; 5 μg/2 μl) into the left SNc and intraperitoneal administration of celastrol (Cel; 1 and 3 mg/kg) on the stereologically estimated number (**a**) and density (**b**) of TH-ir neurons, counted in coronal sections of the rat brain, and the volume of the SN (**c**). Celastrol was administered subchronically for 4 days (1 day before surgery, and then for 3 consecutive days). The rats were killed 7 days after lactacystin administration. Celastrol did not attenuate the dramatic decrease in the number and density of TH-ir neurons on the lesioned side of the SN, induced by lactacystin. The number of animals per group: 5–9. Each bar represents the mean ± SEM. The *symbols* indicate significant differences in the post-hoc test: *p < 0.05, **p < 0.01, ***p < 0.001 versus control group; ^#^ *p* < 0.05 versus Lac group, on the same side of the striatum

Representative photomicrographs of nigral TH-ir neurons from individual treatment groups are shown in Fig. 8. On the lesioned side of the SN, the main effect of lactacystin on the number of TH-ir cells (*F*_1,38_ = 128.84, *p* < 0.001) as well as the main effect of lactacystin and celastrol on the density of TH-ir cells (*F*_1,38_ = 133.64, *p* < 0.001; *F*_2,38_ = 5.08, *p* < 0.05, respectively) were described. As regards the number and the density of TH-ir cells in the intact SN (Fig. 9a, b), only a significant overall effect of celastrol (*F*_2,38_ = 7.66, *p* < 0.01; *F*_1,38_ = 9.77, *p* < 0.001, respectively) was revealed. A post-hoc analysis showed that lactacystin significantly reduced both the number and the density of TH-ir cells in the lesioned SN (Figs. 8e, 5a, b). None of the doses of celastrol (1 and 3 mg/kg) prevented the loss of TH-ir cells or its density in the lesioned SN, when given jointly with lactacystin (Fig. 9a, b). Furthermore, when given alone, the higher celastrol dose reduced the number and/or density of TH-ir cells by *ca*. 30 % on both sides of the SN (Fig. 8c, d). Interestingly, the decrease in the number and density of TH-ir cells induced by celastrol (3 mg/kg) on the intact side of the SN was higher in the lactacystin group (group treated with lactacystin on the opposite side) than in the group treated with celastrol alone (Fig. 9a, b). As regards the volume of the SN, the main effect of lactacystin (*F*_1,38_ = 10.10, *p* < 0.01) was observed on the lesioned side, whereas the main effect of celastrol was revealed on the intact side (*F*_2,38_ = 4.75, *p* < 0.05); however, a post-hoc analysis did not reveal any significant differences in comparison to the control groups (Fig. 9c).

## Discussion

The main finding of the present study is that celastrol does not exert a neuroprotective effect under conditions of UPS inhibition. Furthermore, at higher doses, this compound accelerates toxicity triggered by lactacystin and induces cell death, when given alone in both in vitro and in vivo studies.

Toxicity of lactacystin has been confirmed in many studies using in vitro models (Jantas et al. 2013; McNaught et al. 2002b; Reaney et al. 2006; Rideout et al. 2001). It is generally accepted that the UPS inhibition is the main mechanism responsible for that effect (Fenteany et al. 1995). It was proven that lactacystin at a concentration of 10 μM, hence lower than those used in our experiments, induced a pronounced inhibition (75–90 %) of the chymotrypsin, trypsin-like, and post-glutamyl peptidase activity in PC12 cells (Fornai et al. 2003). Previous studies revealed that suppression of the UPS function by lactacystin led to the accumulation and aggregation of misfolded proteins, and resulted finally in induction of neuronal apoptosis via the release of cytochrome c from mitochondria and the activation of caspase-3-like proteases (Li et al. 2008b; Qiu et al. 2000).

In the present study, we demonstrated, using two different cell cultures (mouse primary cortical neurons and human neuroblastoma RA-SH-SY5Y cells) and a wide range of celastrol concentrations (from 0.01 to 10 μM) that celastrol, given concomitantly with lactacystin, did not protect cells from its toxic effect. Furthermore, we did not find any beneficial effect of celastrol, when that compound was given at various time points (from 1 to 18 h) before lactacystin exposure. The possible explanation for the toxic effect of celastrol in the present study is that this compound, besides its anti-inflammatory and antioxidant properties, is also a potent proteasome inhibitor with preferential inhibition of proteasomal chymotrypsin-like activity (Yang et al. 2006). At a concentration of 2.5 μM, celastrol inhibited chymotrypsin-like activity by *ca*. 40–60 % in purified rabbit 20S proteasome, human cultured prostate tumor cells and Xenopus laevis A6 kidney epithelial cells (Walcott and Heikkila 2010; Yang et al. 2006). At the same concentration, it induced accumulation of ubiquitinated proteins in cells and changes in cell phenotype, accompanied with cytoskeletal disorganization and apoptosis (Walcott and Heikkila 2010; Yang et al. 2006). Hence, it may be speculated that inhibition of the UPS by celastrol may be one of the mechanisms responsible for its toxic effects, especially under conditions of UPS impairment by lactacystin.

Although we found no neuroprotective potency of celastrol, neither given as pretreatment, nor administered jointly with lactacystin, it should be mentioned that effect of celastrol may be biphasic, being protective at low doses and toxic at higher ones. For instance, in PC12 cells, its best protective effect against polyglutamine toxicity or the tert-butyl hydroperoxide (t-BHP)-induced oxidative stress was obtained at 0.1 μM (Sun et al. 2010; Zhang and Sarge 2007), and a toxic effect at 1.6 μM (Sun et al. 2010). In HeLa cells expressing a mutant polyglutamine protein, a significant decrease in cell death was achieved only at concentrations between 0.4 and 1.6 μM (Zhang and Sarge 2007). The neuroprotective effect of low concentrations of celastrol (0.001 and 0.01 μM) was also found in our recent study, but only in cells incubated for longer time in a low-serum medium (Jantas et al. 2013). Therefore, it seems that celastrol has a narrow therapeutic window, and its protective or cytotoxic effect not only depends mainly on its concentration, but also on other factors, e.g., the cell type and culture conditions. However, in the present study, we did not find any protective effects of celastrol against lactacystin-induced toxicity, even when we used the former compound at the low, non-toxic concentrations (0.01 and 0.1 μM). Thus, it appears that under conditions of the strong UPS inhibition by lactacystin, celastrol is not able to protect cells.

Similarly to the above-mentioned neuroprotective effects of celastrol, there are some data demonstrating neuroprotective effect of lactacystin against toxicity induced by low doses of 6-OHDA (Inden et al. 2005; Yamamoto et al. 2007) or by glutamate (Maher 2008; van Leyen et al. 2005). On the other hand, treatment with other UPS inhibitors enhanced 6-OHDA-induced toxicity in different cell cultures (Elkon et al. 2001; Höglinger et al. 2003). These contradictory results may be explained by the fact that 6-OHDA at low doses increases protein degradation and the UPS activity, presumably in response to oxidative stress (Elkon et al. 2001, 2004). In contrast, higher doses cause their marked decline (Elkon et al. 2004). Therefore, it may be speculated that neuroprotection induced by UPS inhibitors is facilitated in those models in which the primary detrimental factor used to destroy neurons is not directly related to UPS inhibition.

On the basis of the above-mentioned studies, it appears that the final effect of the UPS inhibition (cell survival or death) depends strongly on the concentration of the inhibitor used and the duration of its effects (and consequently on the level of UPS inhibition over time). This assumption is consistent with the results of a microarray study (Yew et al. 2005) performed on mouse primary cortical neurons treated with lactacystin that showed different effects at varying post-treatment time points: an up-regulation of genes involved in the neuroprotective response to UPS inhibition at an early time point (e.g., heat-shock protein (HSP) 70, HSP22, genes of the UPS, and cell cycle inhibitors), followed by a proapoptotic response (genes involved in apoptosis, oxidative stress, and inflammatory responses). It has been shown that HSP70 plays a protective role by, for instance, preventing protein misfolding and aggregation (Yenari 2002), and its up-regulation appears to be specific for the cell death mediated by UPS inhibitors (Yew et al. 2005). In fact, celastrol has been found to enhance various HSP levels in vitro; however, the concentrations that enhance HSPs are only slightly lower than the concentrations that are toxic for dopaminergic cells (Chow and Brown 2007). Moreover, in t-BHP-induced cytotoxicity celastrol increases HSP70 expression at concentrations higher than those that are protective in this model (Sun et al. 2010). Therefore, it may be suggested that the increase in HSP70 expression is not a truly neuroprotective property of celastrol, but is rather a result of the defensive reaction of cells against its toxic effect. The above assumption is supported by the fact that the increase in HSP70 content was also observed in surviving dopaminergic neurons of rats treated with a low dose of lactacystin (Pastukhov et al. 2013).

The question arises why primary cortical neurons were more vulnerable to lactacystin toxicity compared to RA-SH-SY5Y cells (2.5 vs. 10 μg/ml, respectively) in the present study. These results are not consistent with the previously described greater sensitivity of various cell lines of tumoral origin to the UPS inhibition compared to normal cells (Adams et al. 1999; Almond and Cohen 2002). These discrepancies may be the result of RA-induced differentiation of SH-SY5Y cells which makes them more resistant to lactacystin toxicity. In fact, a very recent study on SH-SY5Y cells showed a protective effect of RA against the toxic effect of another UPS inhibitor epoxomicin (Cheng et al. 2013). In line with this assumption, we recently found that lactacystin at a dose as low as 0.25 μg/ml decreased cell viability in undifferentiated, but not RA-differentiated, SH-SY5Y cells (Jantas et al. 2013). Likewise, the toxic concentration of celastrol in undifferentiated SH-SY5Y cells was ten times lower than in RA-SH-SY5Y ones (Jantas et al. 2013). Other evidence for the increased resistance of RA-SH-SY5Y cells comes from the present study, namely after treatment of both types of cells with lactacystin and celastrol the increase in cell death was obtained in primary cortical neurons after subtoxic concentration of celastrol, whereas in RA-SH-SY5Y cells a toxic concentration of this compound was required to evoked the same effect.

The lack of the protective effect of celastrol against the lactacystin-induced toxicity in vitro was supported in the present study by utilizing the rat PD model in which degeneration of dopaminergic neurons was caused by unilateral administration of lactacystin directly into the SNc (Lorenc-Koci et al. 2011; Niu et al. 2009; Mackey et al. 2013; McNaught et al. 2002a). The advantage of the lactacystin model over other conventional animal models of PD is related to the fact that it replicates cardinal pathological features of PD, i.e., the nigral degeneration and aberrant protein degradation. We showed that lactacystin (5 μg/2 μl) induced a strong decrease (83 %) in DA level in the lesioned striatum 1 week after surgery, compared to the intact side. Parallel to the change in DA content, a significant decline in the levels of the DA metabolites DOPAC (intraneuronal metabolite), 3-MT (extraneuronal metabolite), and HVA (total metabolite) in the lesioned striatum was observed. Moreover, lactacystin-evoked acceleration of MAO-dependent N-oxidation (DOPAC/DA), COMT-dependent *O*-methylation of DA (3-MT/DA), and the total DA catabolism (HVA/DA) which means that both extraneuronal and intraneuronal DA catabolism were increased after lactacystin administration. The enhanced DA catabolism, especially oxidative catabolism catalyzed by MAO, may at least partly contribute to neuronal death by inducing oxidative stress (Jenner 2003). In fact, there is evidence showing a close mutual relationship between inhibition of the UPS function and oxidative stress (Davies 2001; Lee et al. 2001).

In contrast to the lactacystin group, animals treated with any of celastrol doses showed no decrease in the striatal DA level. There were, however, some differences in the levels of DA metabolites between groups treated with two lower (0.3 and 1 mg/kg) and the highest (3 mg/kg) dose of celastrol. In the former two groups, only a small but significant decrease in the level of DOPAC in the ipsilateral striatum was revealed, whereas in the latter group, DOPAC and HVA levels were elevated, but 3-MT level was decreased in both sides of the striatum. Thus, celastrol seems to influence in the opposite way the intraneuronal DA catabolism, diminishing or increasing it, depending on the dose. This assumption is consistent with the direction of changes in DA catabolic ratios of DOPAC/DA which were decreased after lower doses (the effect more clearly visible on the contralateral side), but tended to rise after the highest dose. Such an increase in the oxidative pathway of DA catabolism may sensitize dopaminergic neurons and make them more vulnerable to lactacystin toxicity. In fact, only the treatment with the highest dose of celastrol accelerated the lactacystin-induced decrease in the striatal level of DA (from 83 to 97 %) and its metabolites compared to the intact side of the striatum. Furthermore, the highest dose of celastrol enhanced the lactacystin-induced acceleration of the MAO-dependent oxidative catabolism and the total catabolism of DA, but it was not accompanied by a simultaneous increase of extraneuronal DA catabolism (3-MT/DA). This implies that exacerbation of DA decline may result from the shifting of DA metabolism from *O*-methylation towards the N-oxidation pathway. On the other hand, in groups treated with lactacystin and the lower celastrol doses, 3-MT/DA ratio substantially increased on the ipsilateral side compared to the lactacystin group. Such enhanced catabolism of DA through COMT-dependent *O*-methylation is supposed to constitute an antioxidant defense mechanism against oxidative stress (Miller et al. 1996). However, since this increase was not accompanied by a substantial increase in DA level, the effect of celastrol seems to be insufficient to overcome the toxicity of lactacystin.

Our biochemical findings were confirmed in the present study by the immunohistochemical data. We demonstrated that lactacystin induced a strong reduction in the number and the density of TH-ir neurons in the lesioned SN one week after surgery. None of the two celastrol doses (1 and 3 mg/kg) prevented the lactacystin-induced loss of nigral TH-ir neurons. Furthermore, the higher celastrol dose was toxic by itself, having reduced the number and density of cells by *ca*. 30 % on both sides of the SN. It is worth noting that the decrease in the number/density of TH-ir neurons on the intact side of the SN in rats treated with a 3 mg/kg of celastrol was greater in the lactacystin group (i.e., the group treated with lactacystin on the opposite side) compared to the rats administered with celastrol alone. The above finding suggests that lactacystin may to some extent spread to the contralateral side of the SN. This assumption is consistent with a most recent study showing a bilateral loss of TH-ir neurons in the SNc and ventral tegmental area, especially after high doses (10 and 20 μg) of unilaterally administrated lactacystin (Mackey et al. 2013). Although the dose of lactacystin we used (5 μg) was too low to damage neurons on the contralateral side of the SN, it could sensitize neurons to celastrol toxicity.

The other finding of our in vivo study was that celastrol, besides induction of degeneration of dopaminergic neurons, may exert systemic toxicity by producing weight loss and mortality in rats. These findings are consistent with studies of Zhu et al. (1996) who revealed toxic effects of celastrol on digestive, urogenital and blood circulatory system in dogs. In our study, only the highest dose of 3 mg/kg of celastrol produced adverse effects and 27 % of rats from this group died before the end of the experiment. Interestingly, a similar dose, given once or twice daily, was found to be effective against MPTP- and 3-nitropropionic acid-induced toxicity in mice and rats, respectively, as well as against the tumor in mice, without inducing toxic side-effects (Cleren et al. 2005; Yang et al. 2006). Moreover, the i.p. LD50 of celastrol for Wistar rats was determined to be much higher (20.5 mg/kg) (Li et al. 2013). On the other hand, one study demonstrated a high mortality (40 %) in mice during celastrol (4 mg/kg) treatment (Raja et al. 2011). Therefore, further studies are required to determinate the safety and tolerability of that compound.

Although in the present study, no beneficial effects of celastrol against lactacystin toxicity were found, it should be noted that some researchers showed neuroprotective effects of that compound in other animal models of PD. For instance, Cleren et al. (2005) demonstrated that celastrol significantly diminished the MPTP-induced loss of dopaminergic neurons in the mouse SN and attenuated striatal DA and DOPAC depletion, probably by attenuation of the MPTP-induced increases in TNF-α and NFκB immunoreactivity, or induction of HSP70. In another study, utilizing a genetic *Drosophila* DJ-IA model, celastrol increased brain DA level and the number of TH-ir neurons in the dorsomedial cluster (Faust et al. 2009). The main reasons for such contrasting results obtained in the present and the above-mentioned studies are most probably the differences in the models of PD which reflect diverse aspects of the pathophysiology of PD and may involve different cellular mechanisms of neurodegeneration. In particular, lactacystin, which is an irreversible UPS inhibitor, induces long-term inhibition of the UPS, since *ca*. 40–50 % decrease in chymotrypsin-like activity can be observed in mouse ventral midbrain (VM) 3–4 weeks after lactacystin administration (Li et al. 2010; Zhu et al. 2007). On the other hand, the *Drosophila* DJ-IA model that refers to an early-onset form of familial PD does not seem to be directly related to UPS dysfunctions (Bonifati et al. 2003; Faust et al. 2009). It has been demonstrated that DJ-1 mutations result in mitochondrial defects and increased neuronal vulnerability to oxidative stress (Takahashi-Niki et al. 2004; Wang et al. 2012). However, DJ-1-deficient mice do not show any UPS dysfunctions, in either the striatum or VM (Yang et al. 2007). The question arises whether MPTP is able to decrease UPS activity in vivo. It has recently been found that only continuous administration of MPTP with an osmotic minipump produces a long-lasting (2 weeks) inhibition of the UPS and formation of inclusion bodies stained for ubiquitin and α-synuclein in the mouse SN. In contrast, conventional MPTP injection induces only a brief UPS inhibition (<24 h) and fails to produce protein aggregation (Fornai et al. 2005). Interestingly, under conditions of conventional MPTP administration, the reversible UPS inhibitor PSI either does not enhance neurodegeneration (Kadoguchi et al. 2008), or even protects dopaminergic neurons against the MPTP-induced toxicity in mice (Oshikawa et al. 2009), thus confirming the earlier observation from in vitro models that the severity and duration of the UPS inhibition may determinate the final, beneficial or adverse effect of the UPS inhibitors.

In conclusion, the presented in vitro and in vivo studies demonstrate, for the first time to our knowledge, that celastrol, a compound which has been shown to be neuroprotective in some models of PD, is no longer protective under conditions of UPS inhibition. Since UPS failure seems to contribute to the pathogenesis of PD, the compounds with a profile similar to celastrol, which, besides anti-inflammatory and antioxidant properties, are also potent proteasome inhibitors, may accelerate the progression of the disease. Therefore, any putative possible pathogenic factors, including those related to the UPS inhibition, must be taken into account in a search for potentially neuroprotective drugs useful in PD treatment strategies.

## Abbreviations

3-MT: 3-Methoxytyramine
DA: Dopamine
DMEM: Dulbecco’s modified Eagle medium
DMSO: Dimethylsulfoxide
DOPAC: 3,4-Dihydroxyphenylacetic acid
FBS: Fetal bovine serum
HPLC: High performance liquid chromatography
HSP: Heat-shock protein
HD: Huntington’s disease
HVA: Homovanillic acid
MAO: Monoamine oxidase
PD: Parkinson’s disease
RA: Retinoic acid
SN: Substantia nigra
SNc: Substantia nigra pars compacta
*t*-BHP: Tert-Butyl hydroperoxide
TH: Tyrosine hydroxylase
TH-ir: TH-immunoreactive
UPS: Ubiquitin–proteasome system
VM: Ventral midbrain
